# Applicability of In Silico New Approach Methods for the Risk Assessment of Tattoo Ink Ingredients

**DOI:** 10.1002/em.70010

**Published:** 2025-05-19

**Authors:** Prachi Pradeep, Stefanie Seifert, Ajay Vikram Singh, Peter Laux, Ralph Pirow

**Affiliations:** ^1^ German Federal Institute for Risk Assessment (BfR) Department of Chemical and Product Safety Berlin Germany

**Keywords:** NAMs, pigments, QSARs, tattoo ink safety

## Abstract

Tattoo inks contain several substances, including organic and inorganic pigments, additives, and solvents, which may pose a health risk to not only the tattooed skin but also to other parts of the human body due to intradermal exposure. Substances in tattoo inks are regulated by entry 75 in Annex XVII of REACH Regulation (EC) No. 1907/2006. However, despite these legal requirements, a well‐defined criterion for the safety assessment of tattoo inks remains lacking. In this context, 2021 BfR opinion titled “Tattoo inks: minimum requirements and test methods” proposed a comprehensive risk assessment of pigments using in vitro/in‐chemico data in accordance with OECD Guidelines and CLP Regulations. In the absence of experimental data, new approach methodologies (NAMs) may be used for data‐gap filling. Therefore, this work evaluates the applicability of in silico NAMs for data‐gap filling for a list of tattoo ink ingredients identified by the Joint Research Centre (JRC) and BfR for genotoxicity assessment. Experimental in vitro genotoxicity data were acquired from the International Uniform Chemical Information Database (IUCLID) which makes non‐confidential REACH Study Results publicly accessible. The specific aims of this analysis were the evaluation of in silico genotoxicity predictions from publicly available QSAR tools and structural alerts, the development and validation of new QSAR models specific to tattoo ink ingredients, and the application of in silico models for categorization and prioritization of data‐poor ingredients for further screening. Based on the workflow developed in this study, 4 high priority, 18 medium priority, and 2 low priority substances were identified for further assessment.

## 1 Introduction

Tattooing is an exemplary art form which continues to gain popularity as a unique form of self‐expression. However, amidst this trend, there are growing concerns regarding the safety and toxicity of tattoo inks on human health. Tattoo inks typically comprise pigments and various additives like carriers, solvents, preservatives, and stabilizers. Pigments provide the vibrant colors and are sourced from diverse materials including metals, organic compounds, or synthetics. Common pigments include coal, iron oxides, and titanium dioxide. Carriers, such as glycerin or water‐based solutions, aid in ink application, while additives like preservatives and stabilizers ensure ink longevity and consistency (Engel et al. 2008; Bäumler 2016; Laux et al. 2016; Breuner et al. 2017; Arl et al. 2019).

The safety profile of tattoo inks raises concerns due to potential risks linked to their ingredients. Heavy metals present as impurities in some pigments, such as chromium VI, nickel, copper, and cobalt, pose significant health hazards, including skin irritation, allergic reactions, and systemic toxicity upon absorption into the bloodstream (Wang et al. 2021). Tattoo inks have also been associated with genotoxic effects. Research indicates that certain components of tattoo inks, such as pigments and additives, may induce DNA damage and genomic instability (Baan et al. 2006; Kluger and Koljonen 2012; Rodrigues‐Lima et al. 2014; Schreiver et al. 2017; Negi et al. 2022). Studies have shown increased levels of DNA strand breaks and oxidative stress markers in individuals with tattoos, suggesting a genotoxic impact of tattoo ink exposure (Schreiver et al. 2017; Hering et al. 2018). Furthermore, the presence of heavy metals, polycyclic aromatic hydrocarbons (PAHs), and aromatic amines in tattoo inks has been linked to mutagenic and carcinogenic properties, raising concerns about long‐term health consequences (Busch and Kuhlencordt 1985; Kluger and Koljonen 2012; Wenzel et al. 2013; Neale et al. 2015; Schreiver et al. 2015; Arl et al. 2019; Kluger 2019a, 2019b; Bäumler 2020; Fraser et al. 2021; Wang et al. 2021; Negi et al. 2022). These findings underscore the importance of further research and regulatory oversight to mitigate the genotoxic risks associated with tattooing.

Tattoo ink regulations vary globally, with some places and organizations imposing strict standards to protect consumers, while others maintain less oversight. In the United States, the Food and Drug Administration (FDA) categorizes tattoo inks as cosmetics, although without mandatory approval requirements (U.S. FDA 2015). Nonetheless, the FDA issues guidance documents outlining best practices for tattoo ink manufacturers to ensure product safety and labeling compliance. In Denmark, the Danish EPA regulates tattooing to ensure safety for artists and clients (Environmental Protection Agency 2014). They establish ink composition standards, restricting harmful substances. Similarly, organizations such as the European Chemicals Agency (ECHA 2023) and the International Agency for Research on Cancer (IARC) offer guidelines and research on tattoo ink safety (Baan et al. 2006). Substances in tattoo inks are regulated by entry 75 in Annex XVII of REACH Regulation (EC) No. 1907/2006 since 2022. Nevertheless, tattoo inks may contain non‐regulated substances. In addition, many substances that are classified by GHS (Globally Harmonized System) were regulated using toxicity data from dermal, oral, or inhalative exposure studies. Toxicity data involving intradermal exposure of these substances are missing.

Despite these legal requirements, a well‐defined criterion for the safety assessment of tattoo inks remains lacking. In this context, the BfR (German Federal Institute for Risk Assessment) opinion titled “Tattoo inks: minimum requirements and test methods” was published in 2021 (BfR 2021). The document proposed a comprehensive risk assessment of pigments using in vitro/in‐chemico data in accordance with OECD guidelines and CLP (Classification, Labeling, and Packaging) regulation. It emphasizes the importance of minimizing toxicological risks associated with tattooing, particularly concerning substances with carcinogenic, mutagenic, or reprotoxic properties, by necessitating the need for comprehensive toxicological evaluations of tattoo ink ingredients, including assessments of potential health effects upon intradermal administration and systemic absorption. However, availability of existing data for toxicological assessment of tattoo ink ingredients varies. While some ingredients have been extensively studied and their adverse effects well‐documented, there are gaps in knowledge for many others. In the absence of experimental data, in silico new approach methods (NAMs) such as quantitative structure activity relationship (QSAR) models are often used for data‐gap filing.

**FIGURE 1:**
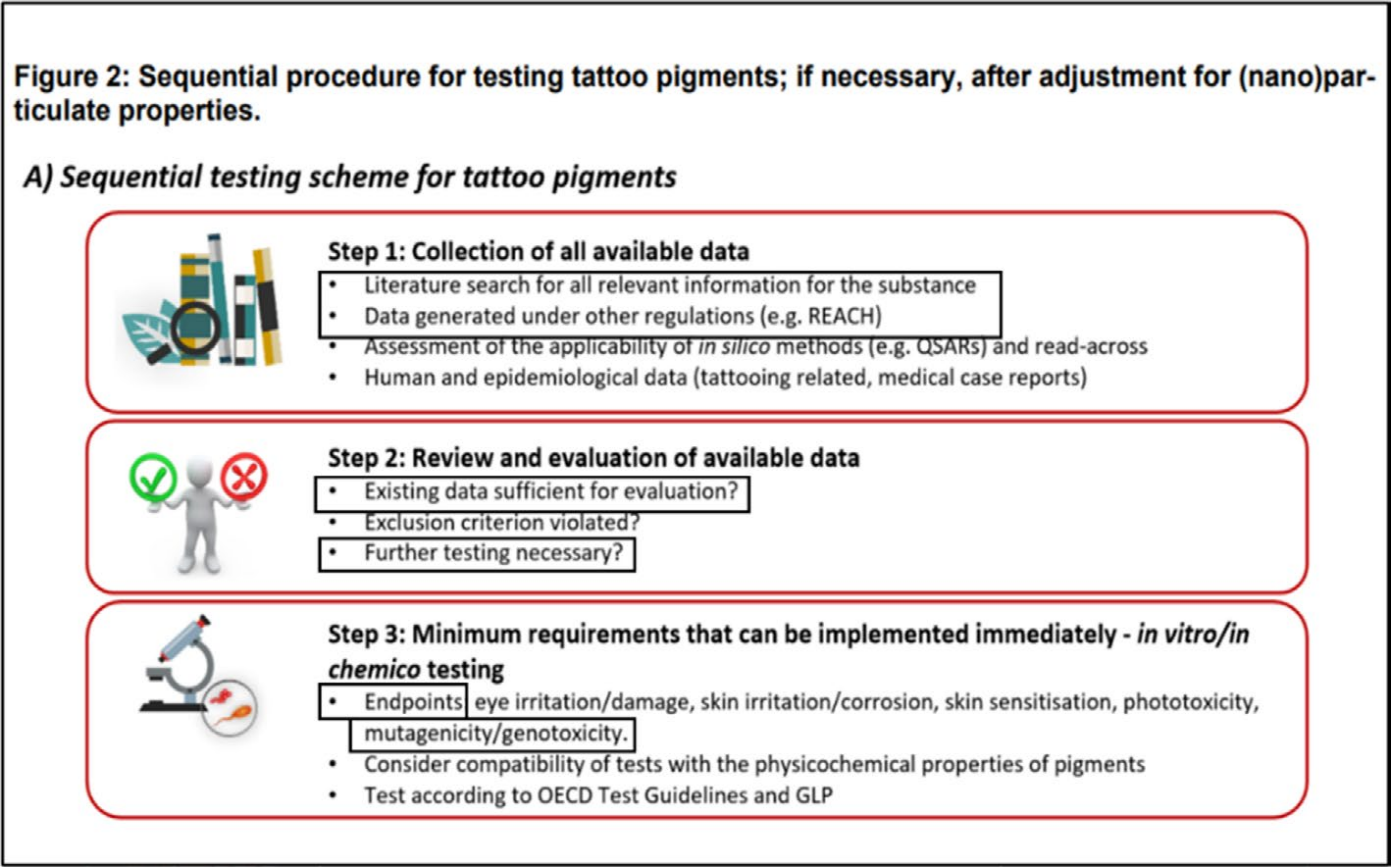
Step‐by‐step procedure that should be followed in the health evaluation of tattoo pigments as described in Figure 2A of the BfR minimum requirements document.

**FIGURE 2:**
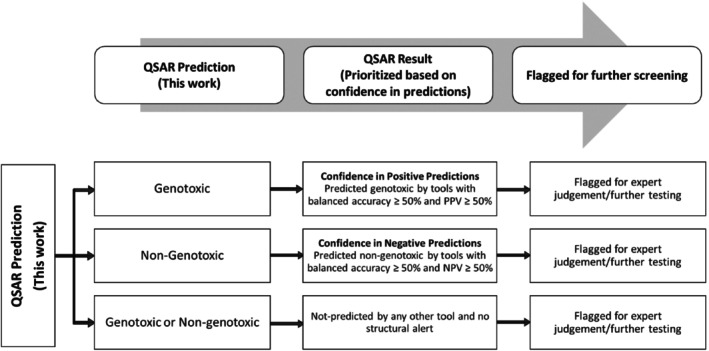
Workflow for prioritizing substances without experimental genotoxicity data for further testing based on QSAR predictions.

The work presented in this manuscript focuses on in silico genotoxicity assessment of tattoo ink ingredients in the context of the BfR minimum requirements. Specifically, the aim of this work is to address the items highlighted in the black rectangular boxes in Figure 1 (a snapshot of Figure 2A from the minimum requirements document that highlights the sequential testing procedure proposed for tattoo ink testing). The overall objectives of the work presented here were:

1. Collection of publicly available REACH submission data for genotoxicity assessment for all substances in the tattoo ink ingredients list originally compiled by JRC and appended to by the BfR with pigments used in tattoo inks.
2. Evaluation of publicly available in silico tools and structural alerts for genotoxicity assessment and their predictive ability for the tattoo ink ingredients.
3. Development of custom QSAR models for predicting genotoxicity of substances in the tattoo inks ingredients list.
4. Development of a screening workflow for the assessment of tattoo ink ingredients lacking experimental genotoxicity data and their prioritization for further investigation.

Overall, this work aimed to create an integrated proof‐of‐concept framework for in silico assessment of tattoo ink ingredients.

## 2 Methods

### 2.1 Dataset and Chemical Structure Information

This analysis relies on a list of regulated ingredients for tattoo ink and permanent makeup, originally compiled by the JRC (Joint Research Centre) (Data S1: 1_tattooList_EPADashboard.csv). The list was subsequently incorporated into Annex XVII of REACH commission regulation (EU) 1907/2006. The amendment of Annex XVII was legally done via the Commission Regulation (EU) 2020/2081 (2020). The utilization of this list is facilitated through the NORMAN Suspect List Exchange initiative, which is a part of the NORMAN network—a collaborative effort involving laboratories, research centers, and related organizations dedicated to monitoring emerging environmental substances (Mohammed Taha et al. 2022; NORMAN Network). This list is available for download from the US Environmental Protection Agency's (EPA) CompTox Chemicals Dashboard (https://comptox.epa.gov/dashboard/chemical‐lists/TATTOOINK). Furthermore, the BfR also consolidated a list of pigments that are used in tattoo ink formulations. This list was compiled from information from ink manufacturers, reports from the German and Australian market surveillance agencies and publications (Commission, Centre et al. 2015; Rigali et al. 2024) (Data S1: 2_tattooListBfR_EPADashboard.csv). For this work, both lists were merged to create a master list referred to as the “tattoo ink ingredients list” hereafter. In all, the master list from both sources comprises 180 unique ingredient substances (Data S1: 3_tattooList_Combined.csv).

The chemical structures for the tattoo ink ingredients list were obtained from the DSSTox database (Richard and Williams 2002; Williams et al. 2017; Grulke et al. 2019) accessible through EPA's CompTox Chemicals Dashboard (Williams et al. 2017) (Data S1: 4_tattooList_WithReachData.smi, 5_tattooList_WithoutReachData.smi). The DSSTox database houses curated chemical structures to ensure correctness in chemical structures and accurate mappings to chemical names and identifiers.

### 2.2 Experimental Data

Experimental in vitro genotoxicity data concerning these substances were acquired from the International Uniform ChemicaL Information Database (IUCLID) (Heidorn et al. 1996) which makes non‐confidential REACH Study Results publicly accessible. The REACH Study Results are a collection of non‐confidential substance data submitted to the European Chemicals Agency (ECHA) under the REACH regulation. The IUCLID REST Web Services API was used within a Python code to access the IUCLID 6 desktop application version 6.27.7 to ensure systematic, automated, and reproducible retrieval of data (Data S1: 6_REACH_data.xlsx).

The experimental study data from IUCLID were reviewed to identify studies which met the following criteria with regards to:

1. Mode of action: The assays were aggregated and classified into three general categories as either Ames or bacterial mutation assay, mammalian clastogen (CA) assay, and mammalian gene mutation assay or other. The classification rules were implemented using the mapping dictionaries defined and made available by (Pradeep et al. 2021). Briefly, these mapping dictionaries were created to standardize terminology and prevent assay misclassification. Assays were then categorized into the above‐mentioned groups. Ames referred to standard bacterial mutation assays using *E* . *coli* WP2 and *Salmonella typhimurium strains*. Clastogen encompassed both in vitro and in vivo chromosomal mutation assays, such as in vitro micronucleus or chromosomal aberration tests, and in vivo micronucleus assays, including the mouse lymphoma test. Gene mutation and other typically included studies based on the OECD TG476 in vitro mammalian gene mutation test using Hprt and xprt genes (2016) and any other study types that were considered non‐standard.
2. Study quality: Only studies with a Klimisch score of 1 (reliable without restriction) and 2 (reliable with restrictions) were included in this analysis. A Klimisch score, developed by Horst J. Klimisch and his colleagues, is a scoring system used in experimental toxicology to assess the reliability of toxicity studies and data (Klimisch et al. 1997).

A final genotoxicity call was derived for each substance based on the experimental data identified. If a substance had one or more positive assay outcomes in any of the three general categories, then it was classified as genotoxic, else it was classified as non‐genotoxic. The final experimental genotoxicity dataset comprised 73 substances from the original 180 substances in the tattoo ink ingredients. The ratio of genotoxic versus non‐genotoxic substances in the final experimental dataset was 21:52 (Data S1: 7_REACH_DataSummary.xlsx).

### 2.3 Genotoxicity Predictions From QSAR Tools and Alerts

Several existing QSAR models and structural alerts were evaluated to assess genotoxicity predictions for the tattoo ink ingredients with experimental genotoxicity data. While there are quite a few models available for assessing mutagenic potential, only a few inform on clastogenic potential. The following tools and alerts (referred together as “tools” hereafter) were evaluated in this study:

1. The TEST tool developed by the US EPA implements several QSAR methodologies to predict mutagenicity, including a hierarchical approach, nearest‐neighbor's approach, and a consensus approach (EPA TEST). Here, we used the consensus approach where the predicted mutagenicity is estimated by taking an average of the predictions from the hierarchical and nearest‐neighbor's methodologies (Martin et al. 2008; Sushko et al. 2010).
2. The VEGA tool is a software platform designed for the prediction of chemical toxicity using Quantitative Structure–Activity Relationship (QSAR) models (Benfenati et al. 2013). VEGA includes genotoxicity models for predicting the Ames test, micronucleus test, comet assay, and chromosome aberration model, which predict a chemical's potential to cause genetic damage. In this work, the VEGA predictions were obtained through the VEGA plugin within the OECD QSAR Toolbox (https://repository.qsartoolbox.org/Tools/Details/0ca4e472‐82a8‐4543‐aeca‐b5a29026d035). The predictions from each of these models are used in a consensus manner to derive a final genotoxicity prediction for each substance i.e., if a substance is predicted positive by any of these models, it is considered genotoxic, else non‐genotoxic.
3. Sarah Nexus is a statistical software developed by Lhasa Limited that uses bacterial reverse mutation test data within fragment‐based structural hypotheses generated from a self‐organizing hypothesis network (SOHN) to make mutagenicity predictions (Sarah Nexus Version 2.5.2 n.d.). In this work, the Sarah Model 2023.1 was used to predict in vitro mutagenicity with weighted reasoning and all default parameters.
4. The OECD QSAR Toolbox is a software tool developed as a collaboration by ECHA designed for use by the government, chemical industry, and other stakeholders to address data gaps in (eco)toxicity. It integrates information and tools from diverse sources into a systematic workflow for easy implementation and access of data. The toolbox also incorporates structural alert schemes referred to as profilers for chemical grouping and categorization. In this analysis, six specific profilers relevant to mutagenicity and clastogenicity were utilized and are listed as follows: (a) DNA alerts for AMES, CA, and MNT by OASIS, (b) DNA binding by OASIS, (c) DNA binding by OECD, (d) protein binding alerts for CA by OASIS, (e) in vitro mutagenicity (Ames test) alerts by the Instituto Superiore di Sanita (ISS), and (f) in vivo mutagenicity (MNT) alerts by ISS. Each of these alerts were treated as an individual source of evidence for predicting mutagenicity. Hence, the presence of an alert in a substance was considered a positive prediction for genotoxicity potential.

A brief summary of all tools is presented in Table 1. The tools were used to make genotoxicity predictions for each substance. The predictive performance of the tools was evaluated using the classification metrics accuracy, sensitivity, specificity, balanced accuracy, positive predictive value (PPV) and negative predictive value (NPV) calculated using the equations listed below:

$$\text{Accuracy}=\frac{\left(\mathrm{TP}+\mathrm{TN}\right)\;}{\left(\mathrm{TP}+\mathrm{TN}+\mathrm{FP}+\mathrm{FN}\right)}$$

$$\text{Sensitivity}=\frac{\mathrm{TP}}{\left(\mathrm{TP}+\mathrm{TN}+\mathrm{FP}+\mathrm{FN}\right)}$$

$$\text{Specificity}=\frac{\mathrm{TN}}{\left(\mathrm{TP}+\mathrm{TN}+\mathrm{FP}+\mathrm{FN}\right)}$$

$$\text{Balanced accuracy}=\frac{\left(\text{Sensitivity}+\text{Specificity}\;\right)\;}{2}$$

$$\text{Positive predictive value}\;\left(\mathrm{PPV}\right)=\frac{\mathrm{TP}}{\left(\mathrm{TP}+\mathrm{FP}\right)}$$

$$\text{Negative predictive value}\;\left(\mathrm{NPV}\right)=\frac{\mathrm{TN}}{\left(\mathrm{TN}+\mathrm{FN}\right)}$$

where TP refers to the total number of true positives, i.e, correctly identified positives (genotoxic substances), TN refers to the total number of true negatives, i.e., correctly identified negatives (non‐genotoxic substances), FP refers to the total number of false positives, i.e., non‐genotoxic substances that were incorrectly classified as genotoxic, and FN refers to the total number of false negatives, i.e., genotoxic substances that were incorrectly classified as non‐genotoxic.

**TABLE 1:** List of all tools (QSAR tools and structural alerts) used for genotoxicity assessment in this study.

In silico tools	Tool type	Details
US EPA Toxicity Estimation Software Tool (TEST) (Version 4.2)	QSAR	Consensus Ames mutagenicity model
VEGA (Version 1.2) (https://repository.qsartoolbox.org/Tools/Details/0ca4e472‐82a8‐4543‐aeca‐b5a29026d035)	QSAR	Consensus mutagenicity model
Sarah Nexus (Version 2.5.2)	QSAR	Ames mutagenicity
OECD Toolbox (Version 4.5)	Structural Alerts	A1: DNA alerts for AMES, CA and MNT by OASIS A2: DNA binding by OASIS A3: DNA binding by OECD A3: Protein binding alerts for Chromosomal aberration by OASIS A4: In vitro mutagenicity (Ames test) alerts by ISS (Istituto Superiore di Sanita) A5: In vivo mutagenicity (Micronucleus) alerts by ISS

### 2.4 Development of New QSAR Models

Validation of existing models showed varying performance (Section 3.1), indicating their reliance on the training dataset (chemical space). The differences in predicted ingredients suggest that current models may not fully capture the chemical space of tattoo inks. Therefore, a custom QSAR model was developed with improved applicability and chemical coverage. The chemical substances used in this analysis were characterized using Toxprints (Yang et al. 2015) and Mordred physicochemical descriptors (https://mordred‐descriptor.github.io/documentation/master/descriptors.html) (Moriwaki et al. 2018). Custom QSAR genotoxicity classification models were developed for tattoo ingredients with experimental genotoxicity data using three different machine learning algorithms—*k*‐nearest neighbor (kNN) (Dasarathy 1991; Altman 1992), support vector classification (SVC) (Cortes and Vapnik 1995; Smola and Schölkopf 2004) and random forest classification (RFC) (Dietterich 2000; Breiman 2001; Shi and Horvath 2006; Kotsiantis et al. 2007; Schapire and Freund 2012). Each algorithm offers a unique approach to classification, with kNN focusing on local instance‐based learning, SVC aiming to maximize class separation (genotoxic versus non‐genotoxic) margins using a non‐linear kernel function, and RFC leveraging the power of ensemble learning through multiple decision trees. A brief description of the molecular descriptors and machine learning algorithms is given in the following subsections.

#### 2.4.1 Molecular Descriptors

Toxprints are a publicly available set of structural features derived from large toxicity databases and regulatory inventories (Yang et al. 2015). These substructures range from an embedded atom, bonds, or chemical groups to structural alerts and Cramer classification rules. Toxprints are 729 bits long, where each bit represents the presence (1) or absence (0) of a structural feature and were accessed through Chemotyper (Chemotyper Application by MN‐AM n.d.), an application developed for the CERES project of the U.S. FDA Center for Food Safety and Applied Nutrition (CFSAN). Mordred descriptors are molecular descriptors used to quantify chemical structures and include over 1800 descriptors including topological, geometric, electronic, and physicochemical properties. These descriptors were generated in the Python programming language using the mordred package (https://github.com/mordred‐descriptor/mordred). Feature selection was carried out on Toxprints and Mordred descriptors using a correlation‐based filter and a variance threshold filter, both set at 70%, to remove highly correlated and low‐variance features. This approach ensures that the selected features contribute meaningful and independent information to the model while reducing redundancy and noise in the dataset. Of the 73 substances with REACH study data, 56 substances could be characterized using these molecular descriptors and were subsequently used to develop the QSAR models.

#### 2.4.2 Machine Learning Algorithms

kNN (Dasarathy 1991; Altman 1992), SVC (Cortes and Vapnik 1995; Smola and Schölkopf 2004) and RFC algorithms (Dietterich 2000; Breiman 2001; Shi and Horvath 2006; Kotsiantis et al. 2007; Schapire and Freund 2012) were used with leave‐one‐out external cross‐validation (LOOCV) and 4‐fold internal cross‐validation. kNN is a non‐parametric, instance‐based algorithm that is similar in concept to read‐across, where for each query instance, a set of neighbors is derived from the training database based on a distance metric. The prediction for the query instance is based on majority voting among the *k*‐nearest neighbors. SVC is a non‐parametric, discriminative algorithm that calculates an optimal hyperplane to create classification boundaries based on the training data. The final prediction is based on the classification boundary, with a default probability cut‐off value of 0.5. RFC is a non‐parametric, ensemble learning algorithm with predictions based on averaging decision trees constructed from training data. RFC is a robust algorithm that reduces overfitting and makes decision tree‐based algorithms more accurate and stable. The final prediction is based on the majority vote from an ensemble of decision trees, with a default probability cut‐off value of 0.5. The training data for the QSAR models comprised 56 substances from the tattoo ingredients list that had experimental REACH study data and chemical descriptors, as described earlier. A complete list of the machine learning algorithms, along with their respective hyperparameters evaluated in this study, is included in Supporting Information (Data S1: 8_QSARSupplementary.docx).

### 2.5 Types of QSAR Models Developed

As described in Section 2.2, each tattoo ink ingredient with experimental genotoxicity data was categorized as Ames, Clastogen, or other. While all these assays assess genotoxicity, the Ames assay focuses on mutations in bacterial test systems, the clastogen assay examines structural changes in chromosomes in mammalian cells and includes any in vitro or in vivo chromosomal mutation assay, and the other category covers mutations and other effects observed in mammalian cell culture (Turkez et al. 2017). Given the different genotoxic modes of action, three types of QSAR models were developed considering individual or overall genotoxic potential:

1. Overall prediction: Models were developed to predict the overall genotoxicity call for each substance, with training data comprising experimental data across all three categories. The final experimental genotoxicity call for each substance was positive if the substance was tested positive in any of the assay categories.
2. Separate predictions: Models were developed to predict whether a substance is positive in any of the categories (Ames, Clastogen, or gene mutation and other). The training data included one to three instances of each substance based on its data across the three assay categories. For example, if Chemical A has data from all three assays—Ames (Positive), Clastogen (Negative), and Other (Positive)—the ‘Overall’ prediction model would consider just one instance, based on its overall genotoxicity result (Genotoxic: Positive). However, in the ‘Separate’ prediction model, Chemical A would contribute three instances—one for each assay outcome.
3. Consensus prediction: Predictions from the separate prediction models were used to derive a final prediction for each substance. The final genotoxicity prediction was positive if a substance was predicted positive in either of the three assay categories. The models were, thus, trained to take the three category predictions and arrive at a final majority consensus prediction.

Predictive performance of the tools was evaluated using the classification metrics accuracy, sensitivity, specificity, balanced accuracy, positive predictive value (PPV) and negative predictive value (NPV) as described in Section 2.3.

### 2.6 Prioritization Workflow

The methods developed here were intended to support screening criteria for data under REACH for tattoo ink ingredients within the context of the BfR minimum requirements proceeding. In that regard, a prioritization workflow was developed where the predictions from the models developed in this work were used together with the predictions from available public tools (Figure 2).

This workflow draws upon the concept of ‘QSAR prediction’ and ‘QSAR result’ as described in the QSAR assessment framework (QAF) document ((Q)SAR Assessment Framework 2023) published by the OECD which is a guidance document for using QSAR model predictions for risk assessment. The predictions from the QSAR model developed in this work is considered the QSAR prediction. In order to combine it with the predictions from the publicly available tools enlisted in Table 1, reference was made to the positive and negative predictive powers of the tools to assign confidence in the predictions. If a substance without experimental genotoxicity data had a positive QSAR prediction, the predictions from the other tools with balanced accuracy ≥ 50% and PPV ≥ 50% were considered. If the substance was predicted positive by these tools, then higher confidence is placed in those positive predictions and the relevant substances were flagged as positive with high confidence. Similarly, if a substance had a negative QSAR prediction, the predictions from the other tools with balanced accuracy ≥ 50% and NPV ≥ 50% were considered. If the substance was predicted negative by these tools, then higher confidence is placed in those negative predictions and the relevant substances were flagged as negative with high confidence. The substances which had no predictions across all tools (including the QSAR model developed herein) were flagged for further evaluation based on lack of supporting evidence. In this way, the QSARs developed in this work are used in conjunction with existing tools to confidently predict genotoxicity for this unique set of ingredients.

## 3 Results and Discussion

### 3.1 Evaluation of QSAR Tools and Alerts

Table 2 presents the performance metrics of in silico tools in predicting the genotoxic potential of substances in the tattoo ink ingredient list. Each approach differs in the underlying methodology and prediction mechanism, leading to differences in the number of ingredients predicted and their respective performance statistics. For example, accuracy ranges from 29.85% to 70.15%, sensitivity ranges from 20.00% to 72.22%, specificity ranges from 12.82% to 91.49%, balanced accuracy ranges from 38.51% to 65.38%, positive predictive value ranges from 23.53% to 50.00%, and negative predictive value ranges from 45.45% to 82.76%. Some approaches demonstrate higher accuracy and sensitivity but lower specificity, while others show the opposite trend. Additionally, the number of ingredients predicted varies slightly among the different approaches, showing the applicability of these tools across different chemical spaces. Overall, these approaches have differences in performance metrics, highlighting the need for careful consideration before use and the need for a custom model that is better applicable to the substances in the tattoo inks.

**TABLE 2:** Performance analysis of existing QSAR tools and structural alerts for genotoxicity prediction of the 73 substances in tattoo ingredients list with experimental data.

Tool	Number of substances predicted/coverage	Accuracy (%)	Sensitivity (%)	Specificity (%)	Balanced accuracy (%)	PPV (%)	NPV (%)
EPA test	47	65.96	58.33	68.57	63.45	38.89	82.76
VEGA	58	31.03	68.42	12.82	40.62	27.66	45.45
Sarah Nexus	59	62.71	72.22	58.54	65.38	43.33	82.76
OECD_A1	67	70.15	20.00	91.49	55.74	50.00	72.88
OECD_A2	67	58.21	20.00	74.47	47.24	25.00	68.63
OECD_A3	67	52.24	55.00	51.06	53.03	32.35	72.73
OECD_A4	67	70.15	20.00	91.49	55.74	50.00	72.88
OECD_A5	67	50.75	55.00	48.94	51.97	31.43	71.88
OECD_A6	67	29.85	60.00	17.02	38.51	23.53	50.00

### 3.2 Evaluation of New QSAR Models

The comprehensive performance metrics for all models are detailed in Supporting Information (Data S1: 8_QSARSupplementary.docx). Considering the overall performance metrics, the Random Forest Classifier (RFC) consensus model emerges as the QSAR model fit for this application. It consistently achieves high accuracy, balanced accuracy, sensitivity, and specificity across different validation methods, indicating its reliability in correctly classifying both positive and negative cases with accuracy 94.6% and balanced accuracy 91.7%.

**FIGURE 3:**
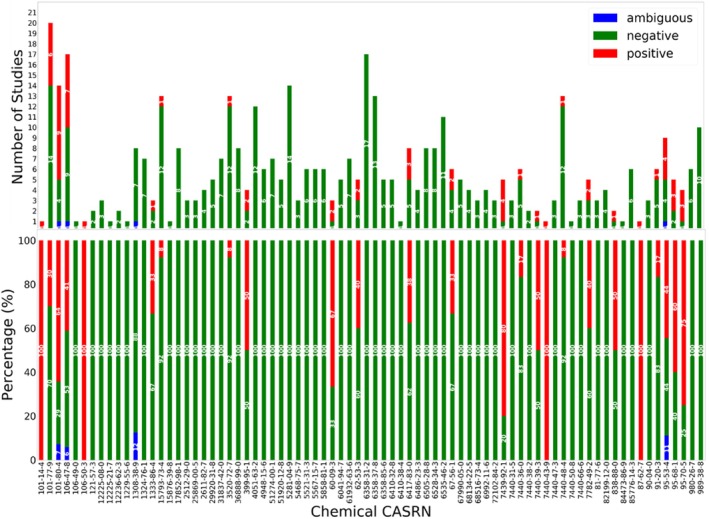
Concordance between experimental study results as obtained from available REACH study data for each tattoo ingredient. Note that 66/78 (~85%) substances have more than 70% agreement in the study outcome.

These results emphasize the significance of both data quantity and quality, with a notable emphasis on the latter for predictive modeling. Despite the small size of the experimental dataset, the variability and quality of the data substantially influenced the performance of machine learning algorithms. As shown in Figure 3, the concordance between study results obtained from the REACH database for each chemical in the tattoo ingredient list shows that about 85% of substances have more than 70% agreement in the study outcomes. This highlights the importance of prioritizing data curation and variability analysis for such studies.

Based on the performance evaluation results (Table 3), the RFC model for making consensus predictions was used as the final model of choice. The added advantage of choosing RFC is the option of feature importance metric computed as the mean decrease in impurity, which quantifies how much each feature (or descriptor) contributes to the overall predictive power of the model. A higher score implies that the feature has a higher influence in the modeled outcome, whereas a lower score indicates lower influence. This helps identify the key factors contributing to the model and understand the importance of each feature in influencing predictions and enhancing model interpretability.

**TABLE 3:** Feature importance calculated as mean decrease in impurity for the final random forest model (consensus prediction).

Descriptor/feature	Mean decrease in impurity	Description
ABC	0.21	Atom‐based connectivity descriptor that quantifies the connectivity of atoms in a molecule by measuring the number of atom pairs connected by specific bond types or distances
jGI4	0.15	2D autocorrelation molecular descriptor that measures the correlation of atomic properties (such as mass or electronegativity) between atom pairs separated by exactly 4 bonds in a molecular graph
jGI5	0.12	2D autocorrelation molecular descriptor that measures the correlation of atomic properties (such as mass or electronegativity) between atom pairs separated by exactly 5 bonds in a molecular graph
AATSC0v	0.12	Averaged centered Broto‐Moreau autocorrelation descriptor that calculates the average of the van der Waals volumes of atoms at a topological distance of 0 (i.e., directly on the atoms themselves)
GATS2v	0.11	2D Geary autocorrelation descriptor that measures the spatial distribution of van der Waals volumes between atom pairs separated by two bonds in a molecular structure
Assay	0.10	Ames, Clastogen or other assay type

Table 3 lists the five most important descriptors in the RFC model along with their computed feature importance in the context of predicting genotoxic potential of tattoo ink ingredients with preexisting experimental data. The ABC descriptor (Apol Barycenter Coordinates) reflects the 3D spatial distribution of atomic partial charges in a molecule. Its relevance to genotoxicity lies in how the charge distribution affects a molecule's interaction with DNA, potentially leading to mutations (Enoch and Cronin 2010; Plošnik et al. 2016; Moriwaki et al. 2018; Khondkaryan et al. 2023); the jGI4 and jGI5 descriptors capture correlations in atomic properties over specific distances, which can indicate reactive sites prone to DNA interaction; the AATSC0v descriptor focuses on atomic charge distributions, essential for predicting electrophilic attack on nucleophilic sites (such as in DNA); the GATS2v descriptor assesses the spatial distribution of van der Waals volumes, revealing potential steric effects that influence reactivity. Additionally, considering the assay type provides insights into the specific genotoxic mechanisms targeted by the tested substances, aiding in predictive modeling. While none of the descriptors can be used to predict genotoxicity individually, together they facilitate the identification of structural motifs associated with genotoxicity.

### 3.3 Prioritization Results

The final RFC model (consensus prediction) and all the tools were used to make genotoxicity predictions for tattoo ink ingredients lacking experimental REACH genotoxicity data (Data S1: 9_Predictions_all.csv). These predictions were then used in accordance with the prioritization workflow outlined in Figure 2.

Substances that were predicted positive by the model were then evaluated for their predictions from the tools that had a balanced accuracy of more than 50% and PPV of more than 50%. Only OECD alerts A1 and A4 qualified as supporting tools in positive outcomes and allowed flagging of four substances for further assessment (Table 4) with high priority. Similarly, the substances that were predicted negative by the model were then evaluated for their predictions from the tools that had a balanced accuracy of more than 50% and NPV of more than 50%. All the tools except VEGA, OECD alert A2, and A6 evaluated in this study qualified to support negative outcomes from the RFC model results and allowed flagging two substances for further assessment with low priority. There were 18 substances that were not predicted by either the RFC model or by the other tools and, hence, were also flagged for further assessment with medium priority (Table 4).

**TABLE 4:** List of all the substances categorized as high, medium, or low concern for further assessment based on the prioritization workflow.

CASRN	Priority	Chemical name
120‐71‐8	High	2‐Methoxy‐5‐methylaniline
95‐80‐7	High	2,4‐Diaminotoluene
106‐49‐0	High	4‐Methylbenzenamine
106‐50‐3	High	1,4‐Benzenediamine
64070‐98‐0	Medium	Methanaminium, N‐[4‐[bis[4‐(dimethylamino)phenyl]methylene]‐2,5‐cyclohexadien‐1‐ylidene]‐N‐methyl‐, molybdatephosphate (1:?)
12768‐78‐4	Medium	C.I. Acid Green 16
1320‐07‐6	Medium	C.I. Acid Orange 24, monosodium salt
1325‐82‐2	Medium	C.I. Pigment Violet 3
12227‐89‐3	Medium	C.I. Pigment Black 11
12769‐96‐9	Medium	C.I. Pigment Violet 15
1309‐37‐1	Medium	Iron oxide (Fe2O3)
1302‐83‐6	Medium	Lazurite
1326‐03‐0	Medium	C.I. Pigment Violet 1
1328‐53‐6	Medium	Pigment Green 7
1332‐37‐2	Medium	Iron oxide
1339‐82‐8	Medium	C.I. Pigment Black 8
1390‐65‐4	Medium	Carmine
15790‐07‐5	Medium	C.I. Food Yellow 3 Aluminum Lake
171599‐85‐2	Medium	N,N′‐Bis(6‐chloro‐4‐(6‐(4‐vinylsulfonylphenylazo)‐2,7‐disulfonicacid 5‐hydroxy‐napht‐4‐ylamino)‐1,3,5‐triazin‐2‐yl)‐N‐(2‐hydroxyethyl)‐ethane‐1,2‐diamine, sodium salt
68921‐42‐6	Medium	FD&C Blue No. 1 aluminum lake
8004‐92‐0	Medium	C.I. Acid Yellow 3 disodium salt
8021‐99‐6	Medium	Charcoal, bone
84632‐59‐7	Low	C.I. Pigment Orange 73
84632‐65‐5	Low	Pigment Red 254

For risk assessment of these substances, a weight of evidence approach is recommended (Hardy et al. 2017). These substances will, therefore, be investigated further to develop a suitable assessment for the use of these substances in tattoo inks. Further investigations may involve literature queries about the legal classification according to the globally harmonized system (GHS).

## 4 Conclusion

The aim of this work was to evaluate the applicability of in silico NAMs to assess genotoxicity for substances relevant to tattoo ink ingredients. Systematic efforts were made to access and leverage REACH study data from the IUCLID public desktop application. These data were used to evaluate existing toxicity assessment models and alerts to determine their suitability for analyzing tattoo ink ingredients. Building upon this information, new custom predictive models were developed employing diverse molecular descriptors and computational techniques to predict genotoxicity. To further improve suitability and acceptability, a screening criterion was devised that allowed prioritization of tattoo ink ingredients for further assessment in a systematic manner based on predictions across different tools. This integrated approach represents a robust proof‐of‐concept for a comprehensive safety assessment framework. By combining data‐driven approaches with expert knowledge and regulatory guidelines, this integrated framework aims to improve the understanding and management of health risks associated with tattooing in the context of the BfR's Tattoo inks minimum requirement's opinion.

## Author Contributions

P.P. and A.V.S. conceptualized the manuscript. P.P. developed the methods, gathered data, performed the analysis, and prepared the manuscript. S.S. assisted in the compilation of the BfR tattoo ingredient list and relevant descriptions for the manuscript. All authors reviewed and approved the final manuscript.

## Conflicts of Interest

The authors declare no conflicts of interest.

## Supporting information


**Data S1.** Supporting Information.

## Data Availability

The data that supports the findings of this study are available in the Supporting Information of this article.
