# Supplementary material for: Applicability of In Silico New Approach Methods for the Risk Assessment of Tattoo Ink Ingredients
Source: Environ Mol Mutagen. 2025 May 19;66(4):199–209. doi: 10.1002/em.70010 (PMC12087733; doi:10.1002/em.70010)
Supplement: Supplementary file 1 — Data S1. Supporting Information. [file EM-66-199-s001.zip › supplemental_revised/8_QSARSupplementary.docx]

**Supplementary Information - QSAR Modeling**

**Supplementary Table 1: A list of machine learning algorithms along with their hyper-parameters evaluated in this work.**

| **Algorithm** | **Hyper-parameters (values) tuned** |
| --- | --- |
| k-nearest Neighbor  (kNN) | **n_neighbors (3-10)**  Number of nearest neighbors to consider.  **weights ('uniform', 'distance')**  'Uniform' gives equal weight to all neighbors; 'distance' gives higher weight to closer neighbors.  **algorithm ('ball_tree', 'kd_tree', 'brute')**  Determines the algorithm used for nearest neighbor search.  **p (1,2)**  Power parameter for the Minkowski distance. 1 = Manhattan distance, 2 = Euclidean distance. |
| Support vector machine (SVC) | **kernel ('rbf')**  Specifies the radial basis function (RBF) kernel for mapping data into higher dimensions.  **C (0.001, 0.01, 0.1, 1)**  Regularization parameter; smaller values allow more margin violations (simpler model), larger values make the model stricter.  **gamma (1, 10, 100)**  Controls the influence of a single training example; higher values mean closer points have more influence.  **decision_function_shape ('ovo', 'ovr')**  Determines multi-class strategy—'ovo' (one-vs-one) or 'ovr' (one-vs-rest).  **random_state (45)**  Ensures reproducibility by setting the random seed |
| Random forest classification (RFC) | **n_estimators (5-10)**  Number of trees in the forest. More trees generally improve performance but increase computation time.  **max_features ('sqrt', 'log2')**  Number of features considered for splitting a node. 'sqrt' (square root of features) and 'log2' (log base 2).  **random_state (45)**  Sets the seed for reproducibility, ensuring consistent results across runs. |

**Supplementary Table 2: Performance Analysis of QSAR Models developed in this work. Considering the overall performance metrics, the Random Forest Classifier (RFC) emerged as the best model.**

| Model | Algorithm | | Number of Substances | | Accuracy  (%) | | Sensitivity  (%) | Specificity  (%) | Balanced Accuracy  (%) | PPV  (%) | NPV  (%) |
| --- | --- | --- | --- | --- | --- | --- | --- | --- | --- | --- | --- |
| Leave-one-out External Cross-validation | | | | | | | | | | | |
| Overall | kNN | 56 | | 92.86 | | 78.57 | | 97.62 | 88.10 | 91.67 | 93.18 |
|  | SVC | 56 | | 73.21 | | 0.0 | | 97.62 | 48.81 | 0.0 | 74.55 |
|  | RFC | 56 | | 85.71 | | 64.29 | | 92.86 | 78.58 | 75.00 | 88.64 |
| Separate | kNN | 179 | | 94.97 | | 82.35 | | 97.93 | 90.14 | 90.32 | 95.95 |
|  | SVC | 179 | | 87.71 | | 38.24 | | 99.31 | 68.78 | 92.86 | 87.27 |
|  | RFC | 179 | | 91.62 | | 73.53 | | 95.86 | 84.70 | 80.65 | 93.92 |
| Consensus | kNN | 56 | | 92.86 | | 78.57 | | 97.62 | 88.10 | 91.67 | 93.18 |
|  | SVC | 56 | | 83.93 | | 42.86 | | 97.62 | 70.24 | 85.71 | 83.67 |
|  | **RFC** | **56** | | **96.43** | | **92.86** | | **97.62** | **95.24** | **92.86** | **97.62** |
